# Antidepressants promote the spread of extracellular antibiotic resistance genes via transformation

**DOI:** 10.1038/s43705-022-00147-y

**Published:** 2022-07-28

**Authors:** Ji Lu, Pengbo Ding, Yue Wang, Jianhua Guo

**Affiliations:** Australian Centre for Water and Environmental Biotechnology (ACWEB, formerly AWMC), The University of Queensland, St. Lucia, QLD 4072 Australia

**Keywords:** Bacteria, Antibiotics, Environmental microbiology

## Abstract

The development of antibiotic resistance as an unavoidable consequence of the application of antimicrobials is a significant concern for human health. Antidepressants are being increasingly consumed globally. Human gut microbial communities are frequently exposed to antidepressants, yet little is known about the interaction between antidepressants and antibiotic resistance. This study aimed to investigate whether antidepressants can accelerate the dissemination of antibiotic resistance by increasing the rate of the horizontal transfer of antibiotic resistance genes (ARGs). Results demonstrated that some of the commonly-prescribed antidepressants (Duloxetine, Sertraline, Fluoxetine and Bupropion) at clinically relevant concentrations can significantly (*n* = 9; *p*_adj_ < 0.01) promote the transformation of extracellular ARGs into *Acinetobacter baylyi* ADP1 for a maximum of 2.3-fold, which is primarily associated with the overproduction of reactive oxygen species. The increased cell membrane permeability and porosity, stimulated transcription and translation of competence, SOS response, universal stress response and ATP synthesis-related genes are also associated with antidepressants-enhanced transformation. This study demonstrated that some antidepressants can speed up the spread of antibiotic resistance by promoting the transformation of ARGs, which emphasizes the necessity to assess the potential risks of antidepressants in spreading antibiotic resistance during clinical antidepressant applications.

## Introduction

The emergence and dissemination of antibiotic resistance have become a global challenge threatening public health, claiming over 1.3 million lives annually in 2019 around the global [1]. In addition to the emergence of antibiotic resistance caused by mutation under antibiotic stress [2], horizontal gene transfer (HGT) greatly contributes to the dissemination of antibiotic resistance [3]. HGT is mediated by three distinct pathways: (i) Conjugation, a physical contact-dependent gene exchange pathway between adjacent bacteria; (ii) Transduction, the bacteriophage-facilitated DNA transfer among restricted host species; and (iii) Transformation, the uptake of exogenous DNA by competent bacteria [4]. Among three HGT pathways, transformation is a unique pathway determined by the chromosomal-encoded competence machinery of the recipient bacteria, which can facilitate the uptake, integration and functionalization of exogenous genetic elements from the surroundings [5], regardless of the existence of conjugal transfer machinery [6]. Under stress conditions (e.g. antibiotics exposure), DNA will be released from the dead or damaged bacteria and can persist in the surrounding *in vivo* or *ex vivo* environments for hours or even months [7]. Therefore, transformation can occur even if antibiotic resistance gene (ARG)-carrying antibiotic-resistant bacteria (ARB) are killed. The released ARGs can still be taken up and integrated by another competent bacterium regardless of their phylogenetic distance [4, 8], leading to the acquisition of ARGs by competent pathogenic bacteria, such as *Staphylococcus aureus, Vibrio cholerae* and *Streptococcus pneumoniae* [9]. Therefore, as one of the predominant pathways for ARG dissemination, transformation is suggested to contribute to the adaptation of human pathogens [10].

It is well recognized that exposure to non-lethal concentrations of antibiotics have accelerated the development and HGT process of antibiotic resistance [11, 12]. Compared to antibiotics, commonly used non-antibiotic pharmaceuticals such as antidepressants are also globally consumed in large quantities, and their prescriptions are surging amid COVID-19 [13, 14]. The half-life of antidepressants in the human body can range from 1 to 2 h for agomelatine, to up to 144 hours for fluoxetine [15] before excretion. Antidepressants are not completely metabolized in the human body and up to 50% (e.g. sertraline) will be discharged through urine or fecal excretion [16]. Therefore, for patients undertaking low-term antidepressants treatment, their gut or urinary microbiome will be constantly exposed to antidepressants of various concentrations. In addition, antidepressants as recalcitrant micropollutants, have been ubiquitously detected in the environment [17]. To date, the role of antidepressants in the spread of antibiotic resistance has been largely overlooked. Recently, an antidepressant-fluoxetine was found to trigger the overproduction of reactive oxygen species (ROS) in *Escherichia coli*, which led to bacterial mutation conferring multiple drug resistance against fluoroquinolone, aminoglycoside, *β*-lactams, tetracycline and chloramphenicol [18]. However, it is not known whether non-antibiotic antidepressants can stimulate the dissemination of ARGs by promoting HGT.

To evaluate the potential roles of antidepressants in prompting the HGT of ARGs, we simultaneously investigated whether commonly-prescribed and widely-detected antidepressants, including Serotonin-norepinephrine Reuptake Inhibitor (SNRI): Duloxetine; Selective Serotonin Reuptake Inhibitor (SSRI): Sertraline, Escitalopram and Fluoxetine; Norepinephrine-dopamine Reuptake Inhibitor (NDRI): Bupropion; and Atypical Antidepressant: Agomelatine, can promote HGT through transformation. A transformation assay was established based on naturally competent recipient bacterium *Acinetobacter baylyi* ADP1 and plasmid pWH1266 which encodes antibiotic resistance against tetracycline and ampicillin. We exposed the transformation assay to six antidepressants at clinically relevant concentrations to test whether the transformation ratio could be significantly enhanced. The underlying mechanisms of promoted transformation ratio were revealed by transformation assays under aerobic or anaerobic conditions, flow cytometry analyses, and genotypic analyses on transcriptional (whole-genome RNA sequencing) and translational (proteomic analysis) responses. Our findings provide insights into the mechanisms that govern HGT associated with antidepressants, in terms of whether and how they can facilitate the spread of antibiotic resistance via transformation.

## Methods

### Bacterial strains, culture media and antidepressants

A transformation assay was established based on naturally competent recipient bacterium *A. baylyi* ADP1 and a competence-deficient mutant *∆comFEBC* strain (*lifO-lipB::aphA3*′*ΔcomFEBC::DHFR-1*) [19]. Plasmid pWH1266 (8.9 kbps, American Type Culture Collection [ATCC] 77092; ATCC, Mansassas, VA, USA) harbouring ARGs against tetracycline (*tetA*) and ampicillin (*bla*_TEM-1_) was chosen as the carrier of extracellular ARGs. *E. coli* DH5*ɑ* cells containing donor plasmid pWH1266 were incubated in LuriaBertani (LB) medium supplemented with 50 mg/L ampicillin shaking (150 rpm) overnight at 37 °C. Invitrogen^TM^ PureLink® Quick Plasmid Miniprep Kit (Life Technologies, USA) was used to extract plasmid pWH1266 from overnight *E. coli* DH5*ɑ* culture.

The recipient *A. baylyi* ADP1 (ATCC 33305) was incubated in LB broth with shaking (150 rpm) overnight at 30 °C to reach the stable phase. Then, 1% of overnight cell culture was transferred into fresh LB broth and further incubated for 6 h at 30 °C with 150 rpm shaking. After, the culture was precipitated by 5 min 6000 × *g* centrifuge and washed twice using phosphate buffer solution (PBS, 137 mM NaCl, 2.7 mM KCl, 10 mM Na_2_HPO_4_, and 1.8 mM KH_2_PO_4_, pH = 7.2). The bacteria were resuspended in PBS containing 40 mg/L sodium acetates as the organic carbon source to reach a OD_600_ of 1.1 for the transformation assay.

All antidepressants including duloxetine, sertraline, escitalopram, fluoxetine, bupropion and agomelatine were purchased from Sigma-Aldrich (USA). Ampicillin was purchased from Gold Biotechnology (USA) and tetracycline was purchased from Sigma-Aldrich (USA).

### Transformation assays

The effects of antidepressants in mediating the transformation of ARGs were evaluated on a model using pWH1266 plasmid as extracellular ARGs, and *A. baylyi* ADP1 as the recipient. In brief, 500 *μ*L transformation assays containing 10^8^ cfu/mL of the recipient (OD_600_ of 1.1) were mixed with donor plasmid to obtain a final concentration of 0.8 ng/*μ*L. Then, duloxetine, sertraline, escitalopram, fluoxetine, bupropion and agomelatine were dosed into each of the transformation assays to reach final concentrations (0, 0.01, 0.1, 1, 10, 50 and 100 mg/L) and incubated without shaking at 25 °C for 6 h (*n* = 9). In order to evaluate the correlations between ROS overgeneration and antidepressants-promoted transformation, two parallel transformation assays as described above with the presence of six antidepressants at designated final concentrations (10 mg/L sertraline; 10 mg/L duloxetine; 10.0 mg/L fluoxetine; 100 mg/L bupropion; 100 mg/L escitalopram and 100 mg/L agomelatine) were individually established with the addition of ROS scavenger thiourea (100 *μ*M) and under anaerobic conditions (*n* = 9). LB agar plates containing 5 *μ*g/mL tetracycline and 100 *μ*g/mL ampicillin were used to quantify the number of transformant cells after a 48 h incubation, while plain LB agar plates were used to quantify the total recipient cell number. The transformation ratio was calculated by dividing the number of transformants by the total number of recipients. The changes in transformation ratios in antidepressant-dosed groups were normalized to the untreated controls to obtain the fold change of transformation ratio.

### Measurements of minimum inhibitory concentration, ROS, and cell membrane permeability

In order to confirm the successful transformation, the IC_90_ against tetracycline and ampicillin of *A. baylyi* recipient and transformants were measured according to a previously described method [20]. The effects of ROS and cell membrane permeability on the transformation ratios were investigated by detecting the ROS production and cell membrane permeability of the *A. baylyi* by a CytoFLEX flow cytometer (Beckman Coulter, USA) in both aerobic (with and without ROS scavenger) and anaerobic mating systems. Briefly, the cellular ROS detection was conducted by using 2’,7’ –dichlorofluorescin diacetate (DCFDA) kit (abcam®, UK), following the manufacture’s protocol. Cell membrane permeabilities were measured by staining with 2 mM of propidium iodide (PI, Life Technologies, USA). A parallel detection was conducted under anaerobic conditions as mentioned above. The fold change of ROS production or cell membrane permeability was calculated by normalizing the ROS level or membrane permeability of antidepressants-treated samples to those of the corresponding untreated control. The test was conducted at least as biological triplicate.

### Live and dead staining assays

To evaluate the inhibitory effects of antidepressants on *A. baylyi*, the percentages of live, damaged, and dead cells after 2 h of various fluoxetine, duloxetine, sertraline, escitalopram, bupropion and agomelatine dosages were quantified by CytoFLEX after duo-stained by LIVE/DEAD^®^ BacLight™ Bacterial Viability Kit (Invitrogen, USA). Detailed testing methods are described in Supporting Information.

### Plasmid extraction, PCR and gel electrophoresis

To confirm the successful uptake of pWH1266, plasmids from randomly selected overnight-cultured transformants or the recipient were extracted and subject to PCR amplification and gel electrophoresis. The *bla*_TEM-1_ gene and *tetA* gene encoded on the pWH1266 plasmid were amplified by using long amplicon PCR primers (Bio-Rad C1000 Touch, USA, Table S1), with an initial 10 min denaturation at 95 °C followed by 30 cycles of amplification at 95 °C with 30 s denaturation, annealing at 52 °C for 30 s and extension at 72 °C for 30 s, ending with a final 5 min extension at 72 °C.

### RNA extraction, genome-wide RNA sequencing and bioinformatics

To investigate the transcriptional response upon antidepressants exposure, transformation assays were established as described above, but in a larger volume (5 mL). Biological triplicated transformation assays containing antidepressant dosages of 0 mg/L (control), 10 mg/L duloxetine, 1 and 10 mg/L sertraline, 50 mg/L escitalopram, 10 mg/L fluoxetine, 100 mg/L bupropion and 50 mg/L agomelatine were incubated for 2 h at 25 °C. Afterward, the total RNA was extracted by the RNeasy Mini Kit (QIAGEN^®^, Germany) and subjected to strand-specific cDNA library construction and Illumina paired-end sequencing (HiSeq 2500, Illumina Inc., San Diego, CA) at Novogene. The bioinformatics pipeline was reported in our previous study [20] and described in the Supporting Information. Differences in gene transcriptional values were calculated between untreated and antidepressant-treated groups by determining the Log2 fold change (LFC) of the averaged fragments per kilobase of a gene per million mapped reads (FPKM) values.

### Protein extraction and proteomic analysis

To investigate the translational response upon antidepressants exposure, another set of transformation assays was established the same as described above in RNA sequencing. After static mating for 4 h, proteins from each antidepressant-treated group (*n* = 3) were extracted using the B-PER method, Digested protein was then analyzed by liquid chromatography (Ultimate^®^ 3000 RSLCnano system, Thermo Scientific^TM^)-tandem mass spectrometry (Q-Exactive^TM^ H-X Hybrid Quadrupole-Orbitrap^TM^ mass spectrometer, Thermo Scientific^TM^) (LC-MS/MS). Raw sequencing data were processed by applying Thermo Proteome Discoverer (version 2.2.0.388) towards the database of *E. coli* MG1655 (received from Uniprot on 12th of July 2019) as described in the Supporting Information. Abundance ratios of each protein between the experimental and the control sample were calculated. A stringency cut-off of false discovery rate (FDR, *q* value) less than 0.01 was applied to identify the proteins with significantly different expression levels.

### Statistical analysis

Data analysis was used by SPSS 27.0 (IBM, Armonk, USA). Significant differences were analysed by Analysis of variance (ANOVA). *p* values were corrected by the Benjamini/Hochberg method. A value of *p*_adj_ < 0.05 was considered significant. Data were expressed as mean ± standard deviation.

## Results

### Antidepressants increased transformation ratio

**Fig. 1:**
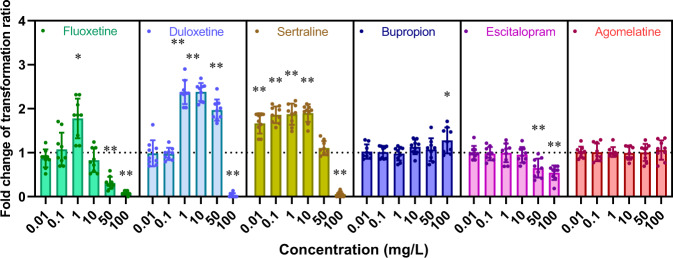
Antidepressants promote the pWH1266 plasmid transformation into *A. baylyi* ADP1. Fold change of transformation ratios of *A. baylyi* ADP1 by plasmid pWH1266 after 6 h treatment of various antidepressants dosages, compared to the untreated control (*n* = 9). Significant differences between individual antidepressant-treated groups and the control groups (0 mg/L) were analysed with ANOVA and shown with * (*p*_adj_ < 0.05), ** (*p*_ad_j < 0.01). *P* values were corrected by the Benjamini/Hochberg method.

Six commonly used antidepressants (duloxetine, sertraline, escitalopram, fluoxetine, bupropion and agomelatine) were tested for their abilities to stimulate the *A. baylyi*’s acquisition of ARGs via transformation, from the concentrations range used in clinical applications (0.01–10 mg/L), to lethal/sub-lethal dosages (duloxetine, sertraline and fluoxetine) against *A. baylyi* ADP1 (50 and 100 mg/L) as confirmed by live/dead staining (Fig. S1) and IC_90_ testing (Table S2) results. For all tested antidepressants, four of them (duloxetine, sertraline, fluoxetine and bupropion) were shown to significantly (*p*_adj_ < 0.05) enhance the transformation ratios of *A. baylyi* ADP1 by ARG-carrying plasmid pWH1266 after 6 h mating. In contrast, all tested dosages of escitalopram and agomelatine could not increase the plasmid uptake ratio of *A. baylyi* ADP1 (Fig. 1 & S2). For duloxetine, sertraline, fluoxetine and bupropion treated groups, the antidepressant-mediated increases in transformation ratio were dosage-independent. The increases in transformation ratio started from 1 mg/L for duloxetine and fluoxetine, 0.01 mg/L for sertraline and 100 mg/L for bupropion, respectively. The maximum increase of transformation ratio reached 2.3-fold (*n* = 9; *p*_adj_ < 0.01) under 1 and 10 mg/L duloxetine exposure, when compared to the spontaneous transformation ratio of the control. By comparison, the transformation ratios of *A. baylyi* ADP1 by plasmid pWH1266 decreased significantly (*n* = 9; *p*_adj_ < 0.01) under the exposure to sub-lethal/lethal dosages of sertraline, fluoxetine and escitalopram (50 and 100 mg/L), as well as duloxetine (100 mg/L) (Fig. 1). These decreases in transformation ratio might be due to the reduced transformants/recipients number under the sub-lethal/lethal dosages of those tested antidepressants (Fig. S1).

To confirm the successful uptake of pWH1266 plasmid, two approaches were applied to examine the existence and the identity of the plasmid in transformants. Firstly, the antibiotic susceptibility results showed the IC_90_ against Amp and Tet for all the transformants were 15 and 5 folds higher than the recipient, which suggested the acquisition of *tetA* and *bla*_TEM-1_ (Table S2). Secondly, plasmid extractions and PCR amplification on *bla*_TEM-1_ and *tetA* genes results showed both donor pWH1266 plasmid and all tested transformants harboured *bla*_TEM-1_ and *tetA* genes, but not recipients (Fig. S3). Collectively, these results confirmed the transformation of pWH1266 plasmid into *A. baylyi* transformants.

### Antidepressants mediate the transformation via inducing oxidative stress

**Fig. 2:**
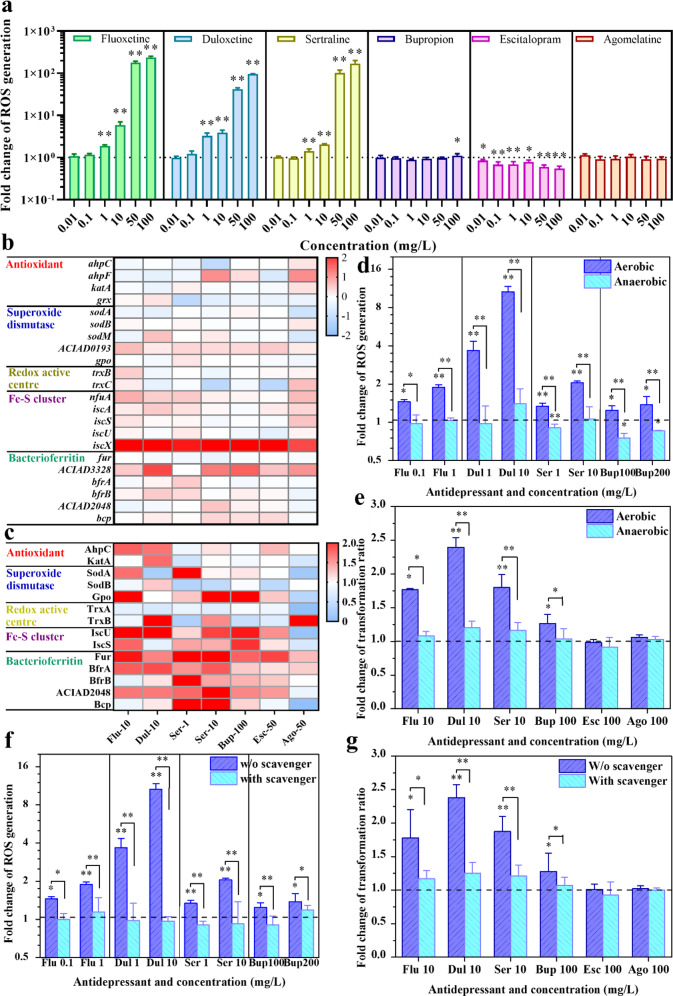
Antidepressants stimulate the ROS generation of *A. baylyi* ADP1. **a** ROS generation of *A. baylyi* ADP1 after 2 h treatments of various antidepressants. Heat-maps showing the transcriptional changes (**b**) and translational changes (**c**) of oxidative stress-related genes under exposure to various antidepressants. Antidepressants-mediated ROS generation (**d**) and transformation of plasmid pWH1266 (**e**) under the anaerobic condition. Antidepressants-mediated ROS generation (**f**) and transformation of plasmid pWH1266 (**g**) with and without the addition of an ROS scavenger-thiourea (100 *μ*M). Significant differences between individual antidepressant-treated groups and the control groups (0 mg/L) were analysed with ANOVA and shown with * (*p*_adj_ < 0.05), ** (*p*_adj_ < 0.01). *P* values were corrected by the Benjamini/Hochberg method.

In order to investigate the mechanisms behind the antidepressant-mediated transformation, the oxidative stress response was monitored under exposure to antidepressants. Firstly, four (duloxetine, sertraline, fluoxetine and bupropion) out of all six tested antidepressants could significantly (*p*_adj_ < 0.05) enhance the generation of ROS in *A. baylyi* ADP1 (Fig. 2a). In contrast, all tested dosages of escitalopram and agomelatine either decreased or could not promote the generation of ROS in *A. baylyi* ADP1 (Fig. 2a). The antidepressant-mediated increases in transformation ratio (Fig. 1) were correlated with the antidepressant-mediated increases of ROS levels (Fig. 2a), when the increase of ROS generation was below a certain threshold (i.e., 50 folds). With the further increases of ROS level to above the threshold (under 50 and 100 mg/L sertraline), the tested antidepressants started to express biocidal effects on *A. baylyi* ADP1, which led to decreases in viable cell numbers (Fig. S1). Subsequently, these biocidal effects might hinder the activities of *A. baylyi* ADP1, thus resulting in reductions in transformation ratios (Fig. 1).

Whole-genome RNA sequencing and proteomic analyses provided further evidence of oxidative stress induced by antidepressants. Under the exposure to different antidepressants, the transcription levels of several genes related to antioxidant (*ahpF*), superoxide dismutase (ACIAD0193) and Fe-S cluster (*nfuA*, *iscA* and *iscX*) and bacterioferritin (*bfrA*, *bfrB* and ACIAD2048) have been significantly (*n* = 3; *p*_adj_ < 0.05) upregulated with a maximum 6.9-fold increase (*iscX*, LFC = 2.8, Fig. 2b). More evidently, the protein translation levels of the majority of these oxidative stress-inducible genes have been upregulated under the exposure to antidepressants, especially for the four antidepressants (duloxetine, sertraline, fluoxetine and bupropion) that were shown to increase ROS generation. In terms of the translation of antioxidants, both duloxetine and fluoxetine promoted the translation of AhpC. In addition, four above-mentioned antidepressants enhanced (i) the translation of either the superoxide dismutase SodAB or Gpo; (ii) the translation of both the Fe-S cluster protein IscU and IscS; and (iii) detoxification proteins Fur, BfrAB,ACIAD2048 and Bcp, with the highest increase of 12.4-fold in IscU translation level under 10 mg/L duloxetine exposure. In contrast, neither escitalopram nor agomelatine could promote the transcription/translation of ROS in *A. baylyi* ADP1 (Fig. 2c).

In order to further validate the causal effects of oxidative stress in mediating the uptake of ARGs by *A. baylyi* ADP1, we conducted an additional transformation assay under both ROS scavenger-added or anaerobic condition where oxidative stress was diminished. Under such conditions, antidepressants could no longer induce ROS-overgeneration. Therefore, the abolishment of increased transformation ratio under such conditions can indicate a causal relationship between ROS-overgeneration and increased transformation ratio. Two parallel transformation assays on four above-mentioned antidepressants were conducted, with one set conducted in an anaerobic chamber (excluding oxidative stress) and the other set dosed with ROS scavenger—thiourea (quenching free radicals) [21], to eliminate the effect of ROS. The ROS detection results showed that both the anaerobic condition (Fig. 2d) and scavenger added groups (Fig. 2f) can significantly (*n* = 9; *p*_adj_ < 0.05) reduce the ROS levels to almost the baseline level of the control group, indicating that all tested antidepressants could not induce excessive oxidative stress to *A. baylyi* ADP1 with scavenger added or under anaerobic conditions. Meanwhile, the results of antidepressant-mediated transformation experiment under anaerobic conditions (Fig. 2e) and with scavenger added (Fig. 2g) showed no noticeable increase in transformation ratio.

### Antidepressants increased cell membrane permeability

**Fig. 3:**
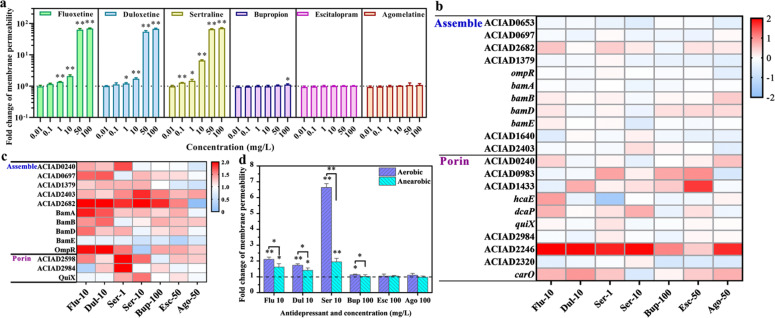
Antidepressants increase the cell membrane permeability of *A. baylyi* ADP1. **a** Cell membrane permeability of *A. baylyi* ADP1 after 6 h treatments of various antidepressants (*n* = 9). Heat-maps showing the transcriptional changes (**b**) and translational changes (**c**) of outer membrane assemble and porin-related genes under the exposure to various antidepressants (*n* = 3). **d** Comparisons between antidepressants-mediated membrane permeability under aerobic and anaerobic conditions (*n* = 9). Significant differences between individual antidepressant-treated groups and the control groups (0 mg/L) were analysed with ANOVA and shown with * (*p*_adj_ < 0.05) or ** (*p*_ad_j < 0.01). *P* values were corrected by the Benjamini/Hochberg method.

The cells at competence state have higher cell membrane permeability and porosity than their non-competent counterparts, which could promoting the uptake of endogenous DNA by crossing the cell outer membrane barrier [22]. In this study, antidepressants were found to enhance the cell membrane permeability and porosity of *A. baylyi* ADP1. Firstly, flow cytometer results showed concentration-dependent increases of membrane permeability on *A. baylyi* under all antidepressants exposure, except bupropion (only 100 mg/L of bupropion increased the membrane permeability) (Fig. 3a). For duloxetine, sertraline, fluoxetine and bupropion-treated groups, the antidepressant-mediated increases of membrane permeability were correlated with the antidepressant-mediated increases in transformation ratio (Fig. 1), when the increase of membrane permeability was below a certain threshold (i.e. 10 folds). In contrast, for escitalopram and agomelatine-treated groups, there was no increase in membrane permeability of *A. baylyi* across all dosages (Fig. 3a), which correspond with no changes in transformation ratio under those antidepressants’ dosage (Fig. 1).

The transcriptional and translational responses of *A. baylyi* ADP1 showed further evidence of antidepressants-promoted membrane permeability. In transcriptional levels, the majority of the porin component genes was upregulated by exposure to antidepressants (Fig. 3b). For example, the transcription of ACIAD2246 gene increased for 5.6-fold (LFC = 2.5) under 10 mg/L fluoxetine exposure. More evidently, at the protein translation level, all the detected outer membrane protein assemble and porin-related proteins (except BamE) have been upregulated under exposure to duloxetine, sertraline, fluoxetine and bupropion, with ACIAD2682 as the most obvious protein (up to 2.4-fold under both 10 mg/L sertraline and 10 mg/L fluoxetine). In contrast, there is no significant change (*p*_adj_ < 0.05) in the translation of outer membrane protein assemble and porin-related proteins under escitalopram and agomelatine exposure (Fig. 3c).

The antidepressants-increased cell membrane permeability and porosity could be associated with the oxidation of membrane lipids due to over-generation of ROS [23]. To investigate the correlation between antidepressant-induced membrane permeability and antidepressant-induced ROS generation, the membrane permeability of *A. baylyi* ADP1 under exposure to antidepressants was tested under anaerobic conditions to ensure the removal of antidepressants-induced ROS. Under anaerobic conditions (Fig. 3d), the membrane permeability of *A. baylyi* ADP1 under all tested antidepressants dosages dropped significantly (*n* = 9; * *p*_adj_ < 0.05, ** *p*_adj_ < 0.01) when compared to those levels under aerobic conditions, to approximately the level of the control group without antidepressant exposure.

### Antidepressants affect the competence-related genes of *A. baylyi*

**Fig. 4:**
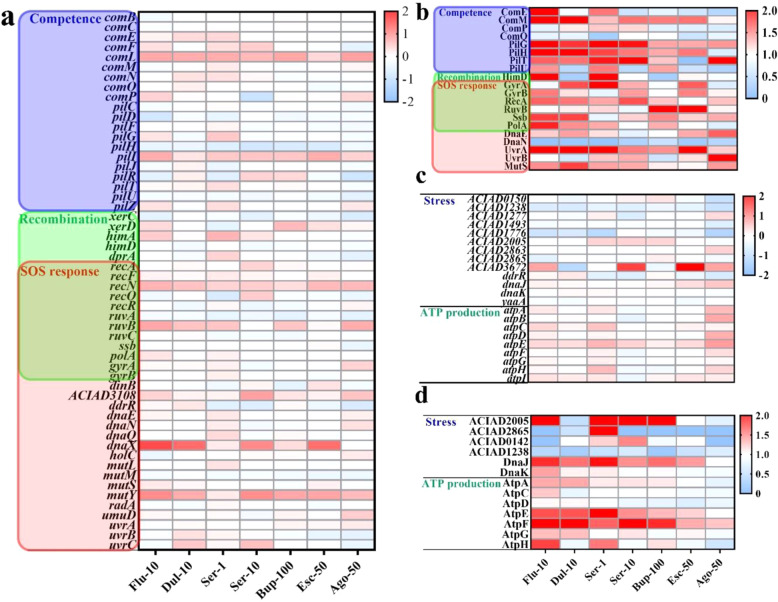
Antidepressants regulate the transcription and translation of competence stress response, recombination, SOS-response and ATP genes. Heat-maps showing the transcriptional changes (**a**) and translational changes (**b**) of competence, recombination and SOS-response related genes; and transcriptional changes (**c**) and translational changes (**d**) of universal stress response and ATP production-related genes under the exposure to various antidepressants (*n* = 3).

In addition to the ROS-overgeneration and increased membrane permeability, the transcriptional and translational changes of competence, recombination and SOS response genes also showed correlations with changes in transformation ratio. To illustrate, the RNA transcription levels of competence-related genes (e.g. *comEFLN*, *pilIR*), recombination genes (e.g. *himA*, *recAFN* and *ruvB*) and SOS response-related genes (e.g. *recAFN*, *ruvB*, ACIAD3108, *dnaX*, *mutY* and *uvrC*) were up-regulated under the exposure to duloxetine, sertraline, fluoxetine and bupropion, while the corresponding up-regulation was limited under escitalopram or agomelatine exposure (Fig. 4a). More specifically, the majority of the protein translation levels of competence (e.g. ComEMP, PilGHTU), recombination (e.g. HimD, GyrAB, RecA, RuvB, Ssb and PolA) and SOS response genes (e.g. GyrAB, RecA, RuvB, Ssb, PolA, DnaE, UvrA and MutS) were enhanced under the exposure to duloxetine, sertraline, fluoxetine and bupropion. Most obviously, the protein translation of two-thirds (14 out of 20 detected protein) of those genes increased under 10 mg/L fluoxetine dosage, with the highest increase of 24.2-fold increase (HimD) (Fig. 4b). In comparison, only 8 out of 20 detected proteins showed enhanced translation (maximum 3.7 fold increase of UvrB) under 50 mg/L agomelatine exposure (Fig. 4b).

Moreover, antidepressants exposure also triggered the universal stress response and ATP production of *A. baylyi*. The transcription and translation levels of some universal stress response-related genes such as GlsB/YeaQ/YmgE (transcription, 3.0 fold under 10 mg/L sertraline, LFC = 1.6), ACIAD2005, DnaK and DnaJ have been up-regulated under the exposure to antidepressants. Meanwhile, the transcription and translation levels SOS response-related genes mostly increased under the exposure to antidepressants, with a maximum increase of 9.9-fold of RuvB protein translation levels when under 100 mg/L bupropion dosage (Fig. 4c, d). In terms of ATP production of, the translations of ATP-related protein were largely enhanced to a maximum of 2.9-fold (Fig. 4c, d) under the exposure to duloxetine, sertraline, fluoxetine and bupropion, which was associated with the increase in transformation ratio (Fig. 1). By contrast, the translations of ATP-related protein were not enhanced by either escitalopram or agomelatine (Fig. 4c, d) that did not promote the transformation of pWH1266 plasmid (Fig. 1).

In order to further evaluate the correlations between antidepressants-induced ROS overgeneration, antidepressants-enhanced membrane permeability and antidepressants-promoted expression of competence during transformation, we used a mutant *∆comFEBC* to conduct the same transformation assay. Results showed that the transformation ratio of *comFEBC*- knocked out *A. baylyi* by pWH1266 plasmid is significantly (*p*_adj_ = 0.0007) three orders of magnitude lower than the wild-type *A. baylyi* (Fig. S4), indicating that *comFEBC* operons play essential roles in the competence of *A. baylyi* species. In addition, despite some antidepressants (duloxetine, sertraline and fluoxetine) significantly increased the ROS generation and cell membrane permeability of *comFEBC*- knocked out *A. baylyi* ADP1 (Fig. S5), all antidepressants-dosed groups did not show significant changes (*p*_adj_ > 0.05) in the transformation ratio of *comFEBC*- knocked out *A. baylyi* by pWH1266 plasmid (Fig. S4). PCR validation was conducted on the total DNA extracted from the recipient *comFEBC*- knocked out *A. baylyi*, transformants and the donor plasmid pWH1266 to prevent false positive (Fig. S6).

## Discussion

### Potential mechanisms of antidepressants-promoted ARGs transformation

In this study, four commonly used antidepressants (duloxetine, sertraline, fluoxetine and bupropion) within the clinical concentration range (0.01 to 10 mg/L) [24], were found to significantly promote the natural transformation ratio of plasmid-borne ARGs into *A. baylyi* ADP1. *A. baylyi* ADP1 is a model for metabolic system biology including natural transformation [25], which shared a highly similar genome with human pathogen *Acinetobacter baumannii* [26]. In addition, *A. baylyi* has also been identified as a pathogen for opportunistic infection [27].

Oxidative stress as one of killing mechanisms of antimicrobials has been associated with the development of antimicrobial resistance [28]. Some antimicrobials generate excessive ROS inside bacteria that can: (i) cause oxidative DNA damage by forming crosslinks or by nucleotide bases modifications, which could result in genetic mutations and the subsequent conversion of bacterial resistance to antimicrobials [29], (ii) cause oxidative cell membrane damage that favours the intake of extracellular components (e.g. ARGs), and (iii) directly regulate the competence of human pathogens, leading to enhanced natural transformation [10, 12]. Therefore, ROS plays an important role in the emergence and spread of antimicrobial resistance.

In this study, we found that the ROS production in the *A. baylyi* ADP1 recipient increased significantly with the increased concentrations of four antidepressants (sertraline, duloxetine, fluoxetine, and bupropion), which is in correspondence with antidepressants-enhanced transformation. The upregulated transcription and translation of antioxidant, superoxide dismutase and redox active centre-related genes as a defense response to oxidative stress [28], further confirmed antidepressants-induced oxidative in *A. baylyi* ADP1. In addition, as indicators against Fe^2+^-induced oxidative damage, the transcription and translations of Fe-S cluster-related genes and ferric uptake regulator (Fur) genes were also provoked under antidepressant exposure. The causation between antidepressants-induced oxidative and antidepressant-mediated transformation was further verified by eliminating the antidepressants-induced oxidative stress, via: (i) a parallel transformation with the addition of a ROS scavenger, and (ii) a parallel transformation experiment conducted under the anaerobic condition to exclude oxygen. Evidently, when the overproduction of ROS is abolished, previously effective antidepressant dosages could no longer enhance the plasmid transformation. Together, flow cytometry ROS detection, RNA sequencing, proteomics and ROS-precluding experiments results indicate that oxidative stress is essential in antidepressant-mediated transformation increase.

In addition, ROS-caused oxidation of membrane lipids may alter membrane functions and increase the permeability of the membrane [23]. A concentration-dependent increase trend in outer membrane permeability of the recipient *A. baylyi* ADP1, and upregulated transcription and translation levels of the outer membrane protein assemble-related genes and porin component genes were observed under the exposure to four antidepressants (duloxetine, sertraline, fluoxetine and bupropion). These increases are associated with the enhancements in antidepressants-induced oxidative stress, upregulated transcription and translation of competence genes and antidepressant-promoted plasmid transformation. When the excessive ROS generation is abolished under the anaerobic condition, both the plasmid transformation ratio and cell membrane permeability return to the control level. Therefore, the enhanced cell membrane permeability was primarily caused by antidepressants-induced oxidative stress.

In order to further evaluate whether ROS-induced membrane damage is essential for the increase in antidepressants-promoted transformation, we further applied a *comFEBC*- knocked out *A. baylyi* to conduct a parallel transformation assay. This mutant strain deleted the active DNA uptake machinery of *A. baylyi* [19], donor DNA would only passively enter the cytoplasm through alternative pores or through damages by diffusion. Firstly, antidepressants were found to significantly increase the ROS production and cell membrane permeability of *∆comFEBC A. baylyi*, which is similar to the response of wild type *A. baylyi*. Secondly, the transformation ratio of *∆comFEBC A. baylyi* by pWH1266 plasmid was significantly three orders of magnitude lower than the wild-type *A. baylyi*, further validating that *comFEBC* operons are essential for the competence of *A. baylyi* species. However, antidepressants-induced cell membrane damages could not promote the transformation of *comFEBC*- knocked out *A. baylyi* by plasmid pWH1266, when the competence machinery is absent. Since ROS overgeneration is the cause of increased membrane permeability, and competent bacteria express a higher membrane permeability for biomolecules like plasmid DNA [5, 22], antidepressants-increased membrane permeability might be the consequence of ROS-overgeneration, or the co-occurrence with the enhanced competence.

During the exponential growth period of competent bacteria, the competence only develops and expresses by a subpopulation of the cells [29, 30]. Upon antidepressants exposure, excessive ROS generation could cause DNA damage [28], which affects the DNA of both the competent and non-competent cell populations of *A. baylyi*. Compared to the non-competent state cells, the competent cells can uptake DNA to repair the ROS-induced DNA damages and they can maintain stationary during the competence state [30], which is similar to the persistent cells under antibiotic stress [31]. In contrast, the non-competent cells are still rapidly growing [30], with insufficient time and DNA materials to repair the ROS-induced DNA damages. Therefore, competence-proficient populations are able to adapt better to the ROS-induced DNA damage and have evolutionary advantages than non-competent cells during active growth [19]. Consequently, under antidepressant stress and the subsequent ROS-induced DNA damage, the better survival of competent bacteria after DNA uptake might promote the transformation.

Although transformation can be promoted under certain antidepressant stress conditions, our results showed that antidepressants at near-IC_90_ did not promote the transformation of plasmid due to excessive damage. Under extensive ROS exposure, lipid peroxidation can cause excessive damage to the properties of the membrane and may lead to the increase of cytoplasmic Ca^2+^ concentration, finally to cell death [23]. Therefore, increased percentages of damaged and dead cells were observed (Fig. S1) under high ROS levels induced by high concentrations of antidepressants (Fig. 2a). As a consequence, the transformation of plasmids was inhibited (Fig. 1) due to excessive cellular damage. Conclusively, antidepressants-induced ROS below a certain threshold can promote the transformation of plasmids.

**Fig. 5:**
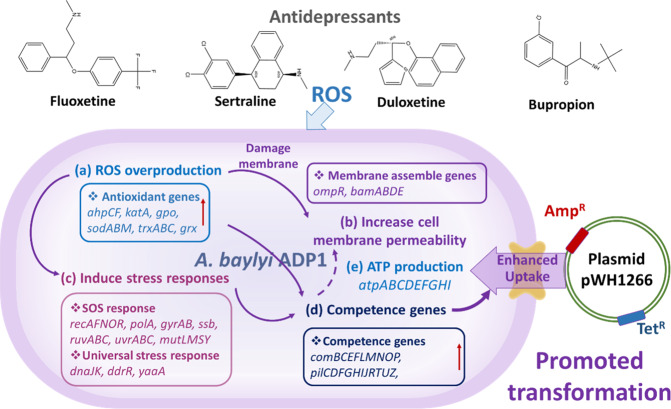
Proposed mechanisms of antidepressants-promoted natural transformation of pWH1266 plasmid into *A. baylyi* ADP1. Firstly, the exposure to antidepressants could: **a** induce ROS overproduction and trigger the expression of corresponding antioxidant-related genes. Consequently, excessive ROS generated by antidepressants can (**b**) damage cell membrane and leads to increased membrane permeability. Spontaneously, oxidative stress induced by antidepressants can (**c**) trigger the SOS and stress response of *A. baylyi* ADP1, thereby promoting (**d**) the expression of competence-related genes. In addition, the elevated competence status might also increase membrane permeability due to the uptake of extracellular materials. Meanwhile, antidepressants also (**e**) promoted ATP production. Conclusively, the serial cellular responses under antidepressants exposure results in the enhanced transformation of *A. baylyi* ADP1 by plasmid pWH1266.

Oxidative stress could also affect the development of competence states in bacteria [32]. The transformation of exogenous DNA into *A. baylyi* consists of three competence pathways: (i) type IV transformation pilus systems (*pil* gene family)-mediated exogenous DNA capture; (ii) DNA translocase complex (*com* gene family)-mediated internalization of single-stranded DNA through the cytoplasmic membrane; and (iii) recombination of exogenous DNA after the homology search [33, 34], which subsequently trigger the SOS response. In this study, the transcription/translation of competence-related genes (Fig. 4a and b) and oxidative stress response-related genes (Fig. 2b, c) increased simultaneously under exposure to duloxetine, sertraline, fluoxetine and bupropion, indicating a possible correlation between oxidative stress and competence status. Moreover, the development of genetic competence has been suggested to be a bacterial surviving strategy in response to stress, which is accomplished by acquiring extracellular DNA for endogenous DNA repair through SOS response signal pathways regulated by *umuD* [35, 36]. The bacterial stressors include but are not limited to oxidative stress, while other stressors such as iron stress, UV stress and antibiotic stress can also trigger the universal stress response of bacteria [12]. Under the exposure to antidepressants, the transcription and translation levels of some universal stress response-related genes have been up-regulated (Fig. 4c, d). Therefore, antidepressants might also facilitate the natural transformation by provoking the stress and SOS response of *A. baylyi*. In addition, our results identified increased transcription and translation levels of ATP-related genes (PilT, PilF, ComEA and ComF-facilitated secretion and DNA internalization) [5, 33] under the exposure to the four antidepressants (duloxetine, sertraline, fluoxetine and bupropion), suggesting the antidepressant-mediated increase ATP synthesis-associated protein translation fuels the transformation process. Collectively, putative mechanisms underlying antidepressant-promoted natural transformation are proposed in Fig. 5.

### Implications to antibiotic resistance and micropollutant management

Our research demonstrated SNRI category antidepressant-duloxetine, SSRI category antidepressant-sertraline and fluoxetine, and NDRI category antidepressant-bupropion at clinical relevant concentration can promote the natural transformation of plasmid-borne ARGs into an opportunistic pathogen *A. baylyi* ADP1. As effective medical interventions, antidepressants are commonly prescribed to treat the major depressive disorder which has the highest regional occurrence rate of 19% [37]. The duration of antidepressant therapy ranges from 6 to 9 months to 3–5 years [38], even possibly an entire lifetime [39] to prevent relapse or recurrence. More importantly, the serious threat to mental health posed by COVID-19 has led to increased antidepressant human consumption [14] and the consequently increased environmental release can be foreseen. Since both the human gut [40] and the environment [4] are known as the reservoirs of ARGs, our findings highlight the potential of antidepressants in mediating the uptake of ARGs by naturally competent bacteria, which is of both clinical and environmental importance.

In clinical aspects, antidepressants can circulate in the human body after being absorbed, with half-life ranging from 1 to 2 h for agomelatine, to up to 144 h for fluoxetine [15] before being excreted through urine or feces. Therefore, the microbiome of patients undertaking antidepressants such as the gut or urinary microbiome will be constantly exposed to various concentrations of antidepressants. In terms of the gut microbiome, antidepressants exposure was associated with alteration in mice gut microbial communities accompanying changes in body weight [41]. More strikingly, several antidepressants including fluoxetine could alter the risk of developing *Clostridium difficile* infection among 4047 adult patients [42]. For the urinary microbiome, a significant association was found between antidepressant use and lower urinary tract symptoms among men aged 45 to 69 years (*n* = 63,579; OR = 1.36, 95% CI 1.29–1.44) [43]. Collectively, our research on the effects of antidepressants in inducing antibiotic resistance via mutagenesis [18], and mediating the spread of antibiotic resistance via promoting transformation of ARGs, have offered insight that antibacterial actions of antidepressants could be associated with antidepressants-induced gut dysbiosis, *C. difficile* infection and urinary microbiome resistome.

Furthermore, in environmental aspects, antidepressants will be released into wastewater after human consumption and are frequently detected in aquatic environments [17, 44]. Wastewater treatment is not completely sufficient in removing antidepressants, around 23% of sertraline still remain after treatment [45]. After being released into the environment, antidepressants such as fluoxetine can be stable for 133 ± 6 days in lake water under sunlight-mediated photodegradation [46]. Since both antidepressants [17, 44–46] and ARGs [4, 7] co-exist and persist in the aquatic environment, antidepressants might pose an unknown risk of mediating the antibiotic resistance dissemination among the environmental microbiome. Consequently, such environmental resistome can be acquired by animals and humans upon exposure [47], thereby posing a substantial risk to the One Health consisted with both human, animal and environmental health. Considering the prevalence of antidepressants in the environment due to extensive consumption, the potential of these non-antibiotic pharmaceuticals in mediating the spread of antibiotic resistance is recommended to be further assessed.

## Conclusions

Conclusively, our research demonstrates that antidepressant drugs can significantly promote horizontal gene transfer via transformation, which are associated with antidepressant-induced ROS overgeneration, membrane damage, SOS and stress response. Our findings offer molecular insights into antibiotic-like properties of antidepressants, and recommend further assessing the risk of spreading antibiotic resistance associated with antidepressant therapies and environmental release.

## Supplementary information

Supporting information

## Data Availability

All data was deposited in publicly accessible databases. RNA sequence data were accessible through Gene Expression Omnibus of NCBI (accession no. GSE181900). The mass spectrometry proteomics data were deposited to the ProteomeXchange Consortium via the PRIDE partner repository (PXD028020).
