# Supplementary material for: Antidepressants promote the spread of extracellular antibiotic resistance genes via transformation
Source: ISME Commun. 2022 Jul 28;2:63. doi: 10.1038/s43705-022-00147-y (PMC9330934; doi:10.1038/s43705-022-00147-y)
Supplement: Supplementary file 1 — Supporting information [file 43705_2022_147_MOESM1_ESM.docx]

**SUPPORTING INFORMATION** for

**Antidepressants Promote the Spread of Extracellular Antibiotic Resistance Genes via Transformation**

Ji Lu^1^, Pengbo Ding^1^, Yue Wang, Jianhua Guo*

*Australian Centre for Water and Environmental Biotechnology (ACWEB, formerly AWMC), The University of Queensland, St. Lucia, Queensland 4072, Australia*

^1^ These authors contributed equally to the work

* Corresponding author. E-mail: [jianhua.guo@uq.edu.au](mailto:jianhua.guo@uq.edu.au) (J. Guo).

**The following are included as supporting information for this paper:**

number of pages: 12

number of figures: 7

number of tables: 2

**SUPPLEMENTARY METHODS**

**Quantification of ROS and Cell Membrane Permeability**

In order to investigate the mechanism of antidepressants-mediated transformation, the detection of ROS and cell membrane permeability were also conducted on a CytoFLEX Flow cytometer according to a previous protocol^9^. Briefly, the cellular ROS detection was conducted by using 2’,7’ –dichlorofluorescin diacetate (DCFDA) kit (abcam®, UK), following the manufacture’s protocol. Cell membrane permeabilities were measured by staining with 2 mM of propidium iodide (PI, Life Technologies, USA). A parallel detection was conducted under anaerobic conditions as mentioned above. The fold change of ROS production or cell membrane permeability was calculated by normalizing the ROS level or membrane permeability of antidepressants-treated samples to those of the corresponding untreated control. The test was conducted at least as biological triplicate.

**Live and Dead Staining Assays**

The inhibitory effects of antidepressants on *A. baylyi* were quantified by profiling the percentage of living, damaged, and dead cells under various antidepressants dosages on a CytoFLEX flow cytometer after duo-stained by SYTO® 9 and PI from LIVE/DEAD® BacLight™ Bacterial Viability Kit (Invitrogen, USA). Stained bacterial samples were then incubated at room temperature in the dark for 30 min. The spectral setting was 488/520 (excitation/emission, in nm) for SYTO® 9, and 488/630 (excitation/emission, in nm) for PI. Untreated and heat-treated (80 °C for 2 h) bacteria were applied as controls for live and dead bacteria, respectively.

**Illumina RNA Sequencing and Global Transcriptional Analysis**

Strand specific cDNA library construction and HiSeq 2500 (Illumina, USA) Illumina paired-end sequencing was conducted by Macrogen (Seoul, Korea). The NGS QC toolkit (version 2.3.3) was used to treat the raw sequence reads to trim the 3’-end residual adaptors and primers, and the ambiguous characters in the reads were removed. Then, the sequence reads consisting of at least 85% bases were progressively trimmed at the 3’-ends until a quality value ≥ 20 were kept. Downstream analyses were performed using the generated clean reads of no shorter than 75 bp. The clean reads of each sample were aligned to the *A. baylyi* reference genome (NC_005966.1) using SeqAlto (version 0.5). Cufflinks (version 2.2.1) was used to calculate the strand-specific coverage for each gene, and to analyze the differential gene expression in triplicate bacterial cell cultures. The statistical analyses and visualization were conducted using CummeRbund package in R (http://compbio.mit.edu/cummeRbund/). Gene expression was calculated as fragments per kilobase of a gene per million mapped reads (FPKM), a normalized value generated from the frequency of detection and the length of a given gene. Differences in fold change values were calculated between 0 mg/L (control), 10 mg/L duloxetine, 1 and 10 mg/L sertraline, 50 mg/L escitalopram, 10 mg/L fluoxetine, 100 mg/L bupropion and 50 mg/L agomelatine mating system by determining the log_2_ fold change (LFC) of the averaged FPKM values of 24, triplicate experiments. Annotation of the differentially expressed genes was based on the online-curated Pathway Tools Genome Database, PseudoCyc (<http://www.pseudomonas.com>).

**SWATH-MS data process and proteomic analysis**

For each sample, 1 *μ*g of the digested protein was used for subsequent SWATH-MS analysis in triplicates. Additionally, 5 μg aliquots of each triplicate sample were pooled for MS library construction by information dependent analysis (IDA), which was performed in duplicate. Peptides were directly analyzed on a Triple-TOF 5600 instrument (Sciex, USA) equipped with a Nanospray III interface. The IDA library and SWATH-MS data were loaded into PeakView v 1.2 software for processing using the SWATH micro-processing script, with a confidence level of 99%, the number of peptides set at five and the number of transitions used set at three. A minimum of two peptides and three transitions was used to determine the abundance of proteins. The R- based program MSstats was used for statistical analysis of the spectral data. Pathway Tools was used for metabolic pathway reconstruction of the identified proteins. Astringency cut-off of with false discovery rate (FDR) value of < 0.05 was used to determine differentially abundant proteins.

**Table S1.** Primers selected for ARGs amplification used in this study.

| Primer | Sequence (5’-3’) | Amplicon Size (bp) | Annealing (℃) |
| --- | --- | --- | --- |
| *blaTEM‑1* long FW | TCC GGT GGA GGC CGG TAT CTG G | 861 | 51.4 |
| *blaTEM‑1* long RV | CGG GAA TGC CAT CTG CCT TGA G |  |  |
| *tetA* long FW | GCTACATCCTGCTTGCCTTC | 1200 | 51.9 |
| *tetA* long RV | CATAGATCGCCGTGA AGAGG |  |  |

**Table S2.** MIC_90_ in mg/L of recipient and transformants (mediated by different antidepressants), against all tested antidepressants, ampicillin (Amp) and tetracycline (Tet).

|  | | Chemical | | | | | | | |
| --- | --- | --- | --- | --- | --- | --- | --- | --- | --- |
|  | | Flu | Dul | Ser | Bup | Esc | Ago | Amp | Tet |
| Organisms | | MIC_90_ (mg/L) | | | | | | | |
| Recipient | | 16 | 8 | 16 | 256 | 256 | 256 | 16 | 2 |
| Transformants mediated by different antidepressants (1 mg/L) | 0 | 16 | 8 | 16 | 256 | 256 | 256 | 128 | 16 |
|  | Flu | 16 | 8 | 16 | 256 | 256 | 256 | 128 | 16 |
|  | Dul | 16 | 8 | 16 | 256 | 256 | 256 | 128 | 16 |
|  | Ser | 16 | 8 | 16 | 256 | 256 | 256 | 128 | 16 |
|  | Bup | 16 | 8 | 16 | 256 | 256 | 256 | 128 | 16 |
|  | Esc | 16 | 8 | 16 | 256 | 256 | 256 | 128 | 16 |
|  | Ago | 16 | 8 | 16 | 256 | 256 | 256 | 128 | 16 |


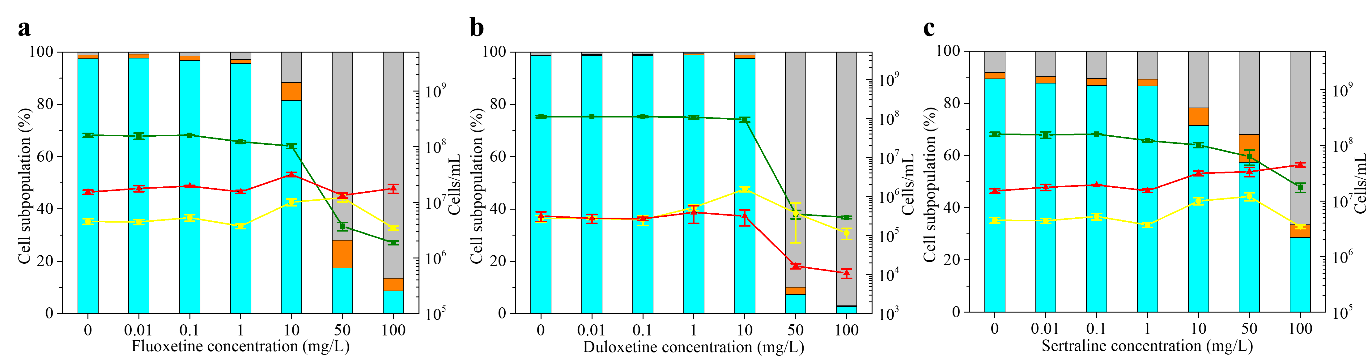


**Fig. S1**. Percentage of live (blue bars), damaged (orange bars) and dead (grey bars) cells and densities of live (green lines), damaged (yellow lines) and dead (red lines) cells under the exposure to different concentrations of (**a**) fluoxetine, (**b**) duloxetine and (**c**) sertraline measured by flow cytometer. Measurements were taken after 2 h antidepressant treatments in PBS at 25 °C. No noticeable changes in live, damaged and dead cells percentages and densities were observed after the treatments of escitalopram, bupropion and agomelatine. Therefore, data of escitalopram, bupropion and agomelatine are not included.

**Fig. S2.** Actual transformation ratios of *A. baylyi* ADP1 by plasmid pWH1266 after 6 h treatment of various antidepressants dosages, compared to the untreated control (n=9). Significant differences between individual antidepressant-treated groups and the control groups (0 mg/L) were analysed with ANOVA and shown with * (*p*_adj_ < 0.05), ** (*p*_ad_j < 0.01). P values were corrected by the Benjamini/Hochberg method.


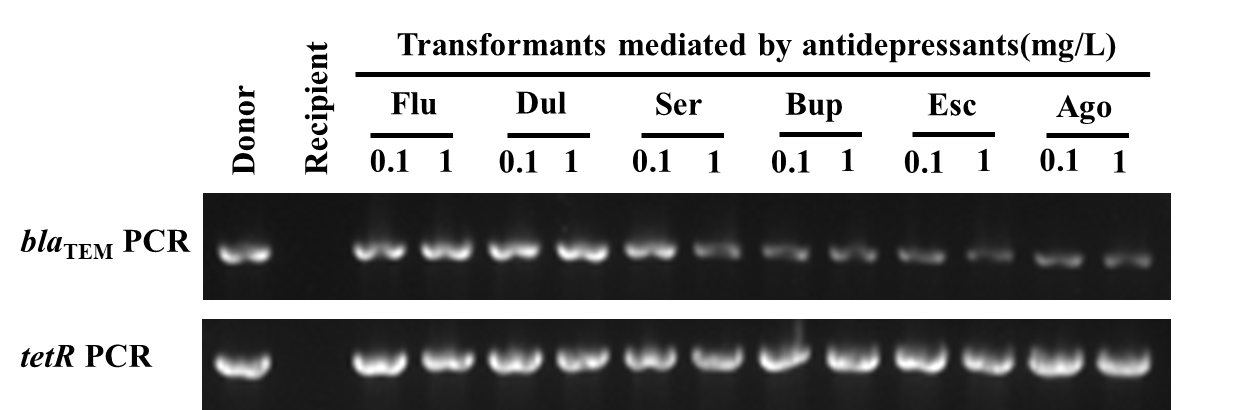


**Fig. S3.** Gel electrophoresis of PCR products on *bla*_TEM-1_ and *tetA* gene on donor pWH1266 plasmid, recipient and transformants*.*

**Fig. S4.** The transformation ratio of wild type and *comFEBC*- knocked out *A. baylyi* by pWH1266 plasmid after 6 h treatment of various antidepressant dosages, compared to the untreated control (n=12). Significant differences between individual antidepressant-treated groups and the control groups (0 mg/L) were analysed with ANOVA and shown with * (*p*_adj_ < 0.05), ** (*p*_ad_j < 0.01). P values were corrected by the Benjamini/Hochberg method.


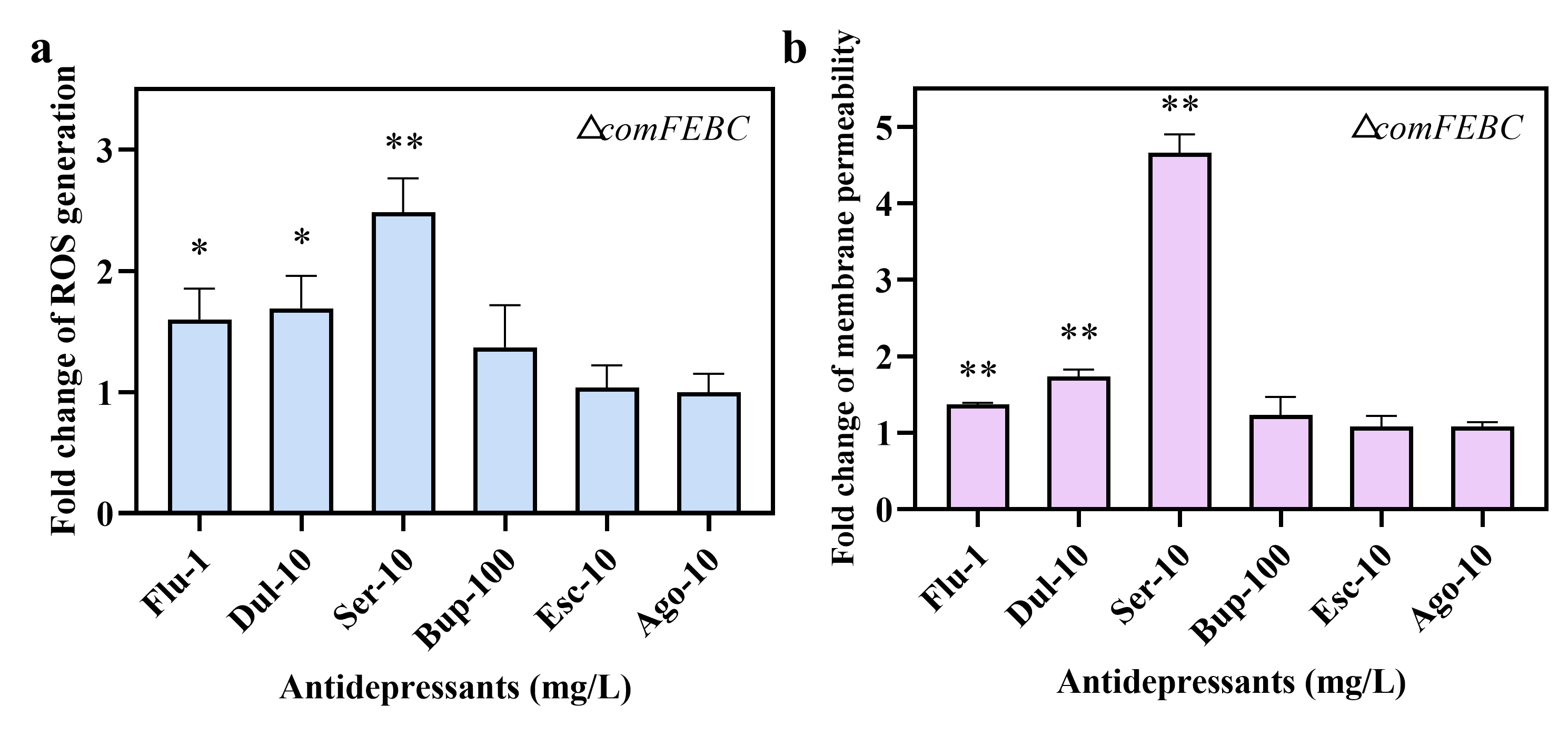


**Fig. S5.** **Antidepressants increase the ROS generation and cell membrane permeability of *comFEBC*- knocked out *A. baylyi* ADP1**. **a**. ROS generation of *comFEBC*- knocked out *A. baylyi* ADP1 after 2 h treatments of various antidepressants. **b.** cell membrane permeability of *A. baylyi* ADP1 after 6 h treatments of various antidepressants (n=4). Significant differences between individual antidepressant-treated groups and the control groups (0 mg/L) were analysed with ANOVA and shown with * (*p*_adj_ < 0.05), ** (*p*_ad_j < 0.01). P values were corrected by the Benjamini/Hochberg method.


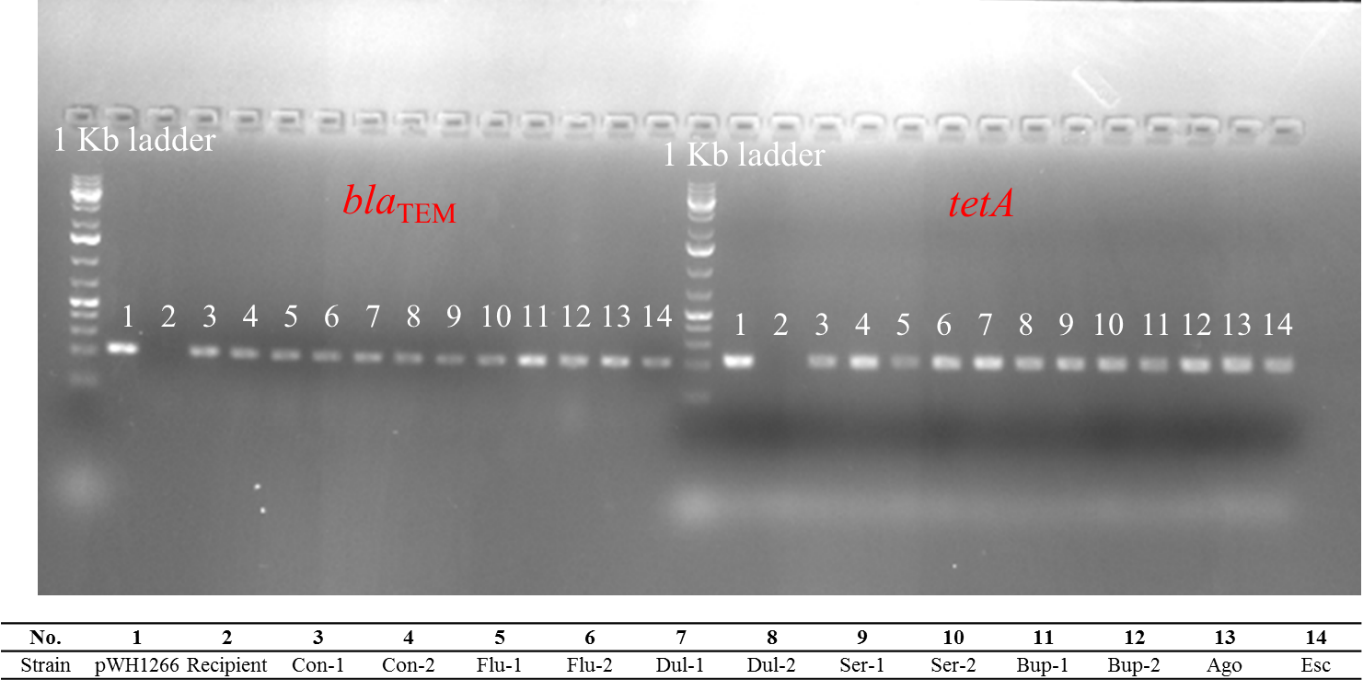


**Fig. S6.** Gel electrophoresis of PCR products on *bla*_TEM-1_ and *tetA* gene on donor pWH1266 plasmid, recipient *comFEBC*- knocked out *A. baylyi* and transformants*.*


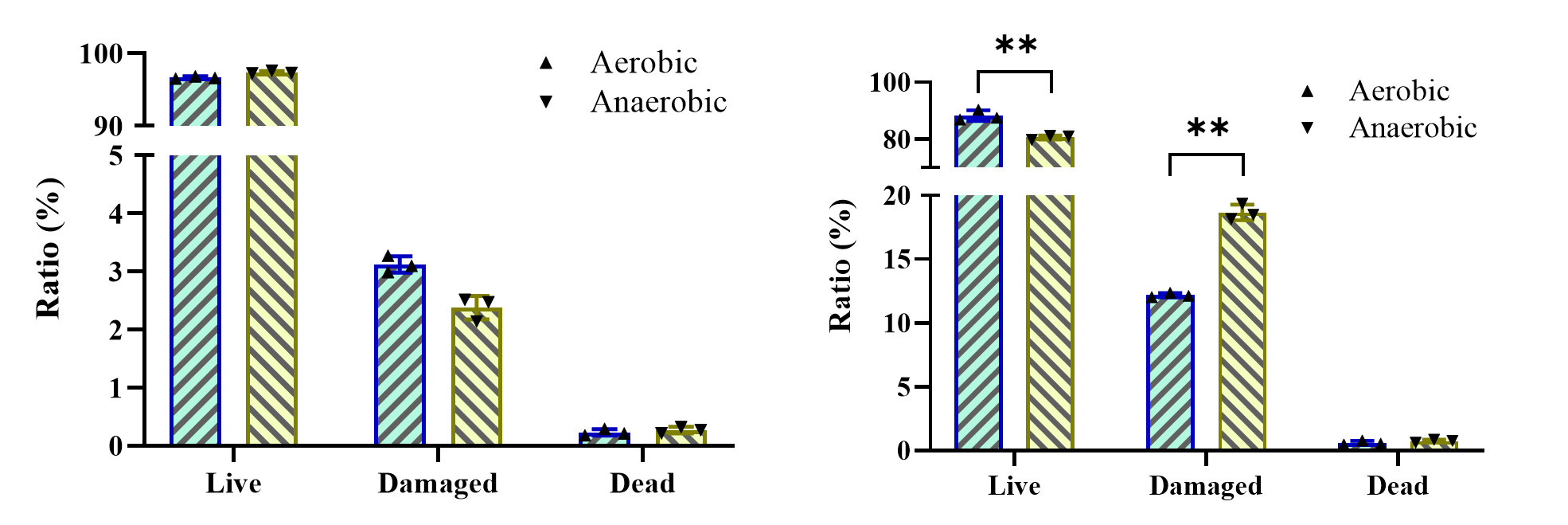


**Fig.S7.** Ratio of live, damaged and dead cells of *A. baylyi* under aerobic (blue) and anaerobic (yellow) conditions for (left) 6 h and (right) 24 h.
